# Synthesis of New Bitopic Tetra(pyrazolyl)-Ligands with Neopentane and O-Xylene Backbones

**DOI:** 10.1100/2012/798271

**Published:** 2012-05-03

**Authors:** Andrei S. Potapov, Evgenia A. Nudnova, Vladimir D. Ogorodnikov, Tatiana V. Petrenko, Andrei I. Khlebnikov

**Affiliations:** ^1^Department of Chemistry, Altai State Technical University, 46 Lenin Street, Barnaul 656038, Russia; ^2^The Laboratory of Physicochemical Methods of Analysis, Institute of Petroleum Chemistry, Siberian Branch of Russian Academy of Sciences, 3 Akademicheskii Avenue, Tomsk 634055, Russia

## Abstract

Several new bitopic pyrazole-containing ligands were prepared from the corresponding pyrazoles and tetrahalogen or tetratosyloxy derivatives of o-xylene and neopentane in a superbasic medium (KOH-DMSO).

## 1. Introduction

**Figure 1:**
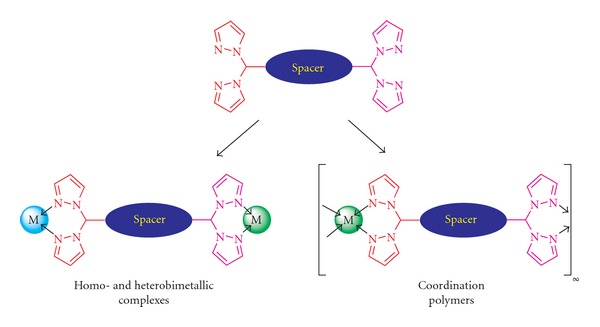
Structure of bitopic ligands with bis(pyrazol-1-yl)methane units and their two possible coordination modes.

Bitopic ligands are compounds possessing two separate metal binding cites [1]. These ligands can act as building blocks for syntheses of homo- and heterobimetallic coordination compounds, as well as coordination polymers. These types of compounds are interesting due to their potential catalytic activity and diverse supramolecular architecture (Figure 1).

 Ligands bearing two bis(pyrazol-1-yl)methane units linked by aliphatic and aromatic spacers were first prepared by Daniel Reger and are referred to as the third-generation scorpionates [2].

In this communication we report the synthesis of two new types of bis(pyrazol-1-yl)alkane bitopic ligands. In one of them two 1,3-bis(pyrazol-1-yl)propane moieties are linked together directly without a spacer. In the other ligand two bis(pyrazol-1-yl)methane units are linked by an *ortho*-phenylene spacer forming a previously unavailable sterically hindered compound.

## 2. Materials and Methods

Elemental analyses were carried out on a Carlo Erba analyzer. NMR spectra were recorded on Bruker AV300 instrument operating at 300 MHz for ^1^H and 75 MHz for ^13^C. EI MS measurements were carried out using TRACE DSQ (Thermo Electron Corporation, USA) instrument.

DFT calculations were carried out at RI DFT BP86 [3] level of theory and TZVPP [4] basis set (TZV/J auxiliary basis set [5, 6]) using ORCA 2.8.0.2 package [7].

1,3-Dibromo-2,2-bis(bromomethyl)propane [8] and 1,2-bis(dibromomethyl)benzene [9] were prepared using literature methods. Pentaerythritol tetratosylate was prepared from pentaerythritol and p-toluene sulfochloride in acetone-aqueous NaOH by adopting a procedure from [10].

**Tetrakis[(pyrazol-1-yl)methyl]methane (1)**

A suspension of 0.5 g (7.35 mmol) of pyrazole, 0.823 g (14.7 mmol) of powdered KOH in 7 mL of DMSO was stirred at 80°C for 30 minutes. After that, 0.714 g (1.84 mmol) of 1,3-dibromo-2,2-bis(bromomethyl)propane were added in three equal portions every 30 minutes. Stirring was continued for 24 hours at 80°C, then 70 mL of water were added, the solution was neutralized with hydrochloric acid and extracted with chloroform (5 × 10 mL). The extract was washed with water (2 × 10 mL), dried over calcium chloride and evaporated in vacuo. Product yield 0.224 g (36%), colorless crystals, m.p. 192–194°C (EtOH). NMR ^1^H (CDCl_3_), *δ*, ppm: 4.23 (s, 8H, CH_2_), 6.27 (t, 4H, J = 1.8 Hz, H^4^), 7.58 (d, 4H, J = 1.8 Hz, H^3^), 7.76 (d, 4H, J = 1.8 Hz, H^5^). NMR ^13^C (CDCl_3_), *δ*, ppm: 45.9 (C(CH_2_)_4_), 52.1 (CH_2_), 105.3 (C^4^-Pz), 132.4 (C^5^-Pz), 139.9 (C^3^-Pz). Anal. found, %: C 60.32; H 5.90; N 33.04. C_17_H_20_N_8_. Calculated, %: C 60.70; H 5.99; N 33.31.

**3,3-Bis(pyrazol-1ylmethyl)oxetane (2)**

A suspension of 0.5 g (7.35 mmol) of pyrazole, 0.823 g (14.7 mmol) of powdered KOH in 5 mL of DMSO was stirred at 80°C for 30 minutes. After that, 1.38 g (1.84 mmol) of pentaerythritol tetratosylate were added in three equal portions every 30 minutes. Stirring was continued for 24 hours at 80°C, then 50 mL of water were added, the solution was neutralized with hydrochloric acid and extracted with chloroform (5 × 10 mL). The extract was washed with water (2 × 10 mL), dried over calcium chloride, and evaporated in vacuo. Product yield 0.315 g (79%), colorless oil. NMR ^1^H (CDCl_3_), *δ*, ppm: 4.30 (s, 4H, CH_2_–O), 4.67 (s, 4H, CH_2_–Pz), 6.22 (t, 4H, J = 1.5 Hz, H*^4^*), 7.58 (d, 4H, J = 1.5 Hz, H*^3^*), 7.76 (d, 4H, J = 1.5 Hz, H*^5^*). NMR ^13^C (CDCl_3_), *δ*, ppm: 45.0 (C(CH_2_)_4_), 54.0 (CH_2_–O), 77.4 (CH_2_–Pz), 105.1 (C^4^-Pz), 130.8 (C^5^-Pz), 139.9 (C^3^-Pz).

**3,3-Bis(3,5-dimethylpyrazol-1-ylmethyl)oxetane (3)**

It was prepared similarly to compound **2** from 0.5 g (5.21 mmol) of 3,5-dimethylpyrazole, 0.98 g (1.30 mmol) of pentaerythritol tetratosylate, and 0.58 g (10.4 mmol) of KOH in 5 mL of DMSO. Yield 0.239 g (67%), colorless crystals, 98–100°C. NMR ^1^H (CDCl_3_), *δ*, ppm: 1.86 (s, 6H, 3-CH_3_), 2.17 (s, 6H, 5-CH_3_), 4.14 (s, 4H, CH_2_–O), 4.79 (s, 4H, CH_2_–Pz), 5.72 (s, 2H, H*^4^*). NMR ^13^C (CDCl_3_), *δ*, ppm: 10.4 (5-CH_3_-Pz), 13.5 (3-CH_3_-Pz), 44.8 (C(CH_2_)_4_), 50.0 (CH_2_–O), 78.6 (CH_2_–Pz), 104.4 (C^4^-Pz), 139.9 (C^5^-Pz), 147.7 (C^3^-Pz). MS (EI, 70 eV), m/z (I, %): 274 (2%, [M]^+^), 244 (55%, [M-2CH_3_]^+^), 165 (48%, [M-PzCH_2_]^+^), 109 (100%, [PzCH_2_]^+^).

**1,2-Bis[bis(pyrazol-1-yl)methyl]benzene (4)**

It was prepared similarly to compound **1** from 0.5 g (7.35 mmol) of pyrazole, 0.82 g (14.7 mmol) of KOH, and 0.78 g (1.84 mmol) 1,2-bis(dibromomethyl)benzene in 10 mL of DMSO, reaction duration 7 hours. Yield 0.377 g (55%), colorless crystals, m.p. 98–99°C (i-PrOH). NMR ^1^H (CDCl_3_), *δ*, ppm: 6.35 (t, 4H, J = 2 Hz, H*^4^*-Pz), 6.63 (d, 2H, J = 3 Hz, H*^3^*-Ph), 7.44 (d, 2H, J = 3 Hz, H*^4^*-Ph), 7.59 (d, 4H, J = 2 Hz, H*^3^*-Pz), 7.63 (s, 2H, Pz_2_CH), 7.68 (d, 4H, J = 2 Hz, H*^5^*-Pz). Anal. found, %: C 64.42; H 4.58; N 29.80. C_20_H_18_N_8_. Calculated, %: C 64.85; H 4.90; N 30.25.

**1,2-Bis[bis(4-iodopyrazol-1-yl)methyl]benzene (5)**

A solution of 0.1 g (0.270 mmol) of compound **4**, 0.11 g (0.432 mmol) of iodine, 0.19 g (1.08 mmol) of HIO_3_ in 5 mL of dioxane, and 2.5 mL of water was refluxed for 5 hours. After cooling to room temperature, the precipitate was formed, which was filtered and washed with water. Yield 0.152 g (64%), colorless crystals, m.p. 180°C (decomposed, dioxane-water, 3 : 1). NMR ^1^H (DMSO-*d *_6_), *δ*, ppm: 6.96 (2H, H^3^-Ph), 7.61 (s, 4H, H*^3^*-Pz), 7.75 (s, 4H, H*^5^*-Pz), 7.85 (2H, H*^5^*-Ph),7.91 (s, 2H, Pz_2_CH).

## 3. Results and Discussion

**Scheme 1:**
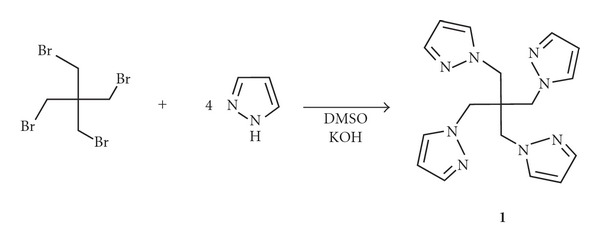
Synthesis of tetrakis[(pyrazol-1-yl)methyl]methane **1**.

Previously unknown bitopic ligand **1** was prepared by the reaction of 4 equivalents of pyrazole (PzH) with neopentane tetrabromo derivative (1,3-dibromo-2,2-bis(bromomethyl)propane) (NTB) in a superbasic KOH-DMSO medium (Scheme 1). The moderate yield of 36% could not be improved by varying the reagents ratio (PzH : NTB : KOH) or reaction temperature (from 20 to 120°C), which is probably due to steric reasons. In agreement with this, more bulky 3,5-dimethylpyrazole did not give substitution product at all and only starting materials were recovered.

**Scheme 2:**
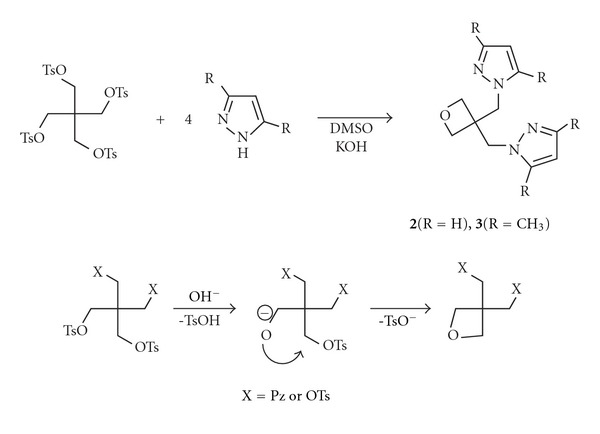
Formation of 3,3-bis(pyrazol-1-ylmethyl)oxetanes **2**, **3** as a result of intermolecular cyclization.

In an attempt to improve product yield we tried to change the bromo leaving group to tosyloxy moiety. Unexpectedly, no tetra-pyrazolyl substituted product was obtained in this case. Instead, bis(pyrazolylmethyl) derivatives of oxetane were obtained, apparently as a result of intramolecular cyclization (Scheme 2). Similar cyclizations under the action of strong bases were reported previously for tri- and tetratosylates of pentaerythritol [11].

Synthesis of unsubstituted pyrazole compound **2** from potassium pyrazolidine and corresponding 3,3-dimethyloxetane derivative has been reported earlier [12]. The method of synthesis proposed here is more convenient and allowed to prepare previously unknown dimethyl derivative **3**.

Compounds **2** and **3** are interesting as ligands for coordination chemistry and as semiproducts for synthetic organic chemistry, since the reactive oxetane cycle can be opened by different basic agents with the formation of a variety of polyfunctional compounds.

**Scheme 3:**
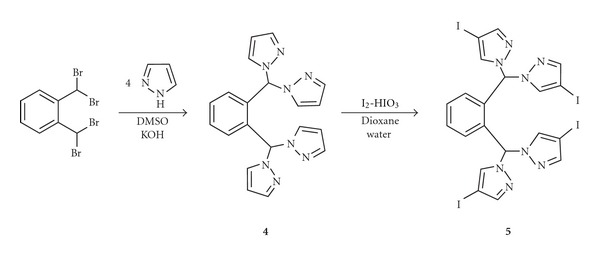
Oxidative iodination of 1,2-bis[bis(pyrazol-1-yl)methyl]benzene.

We have also prepared a new bitopic ligand with o-phenylene spacer. Compound **4** was obtained by the reaction of *α*, *α*, *α*′, *α*′-tetrabromo-o-xylene with pyrazole in a superbasic medium (Scheme 3). Previously we have used this method for the preparation of ligands with p-phenylene spacer [13].

**Figure 2:**
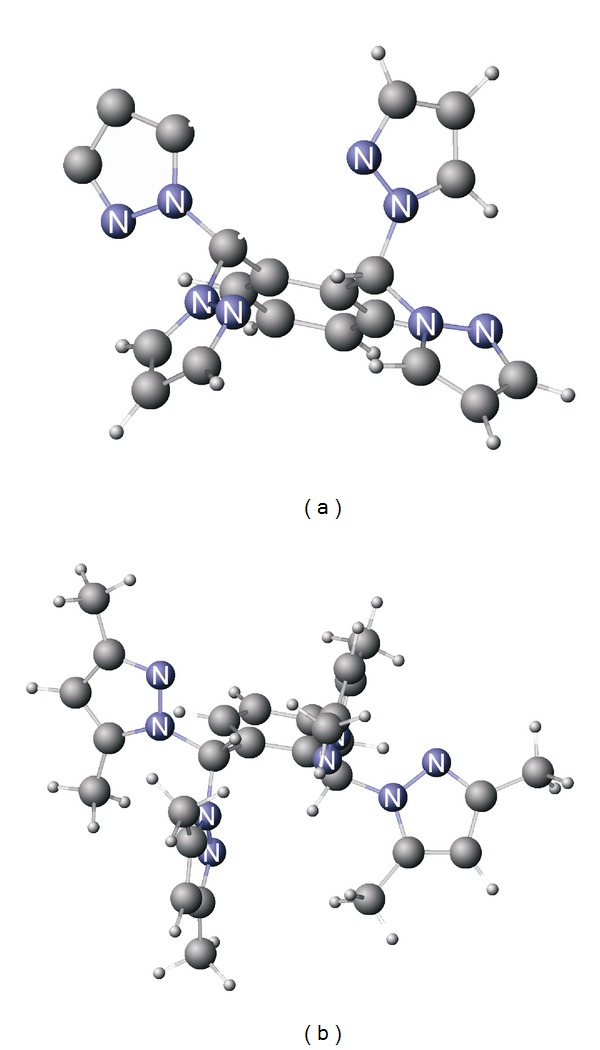
Molecular models of compound **4** (a) and its dimethylsubstituted derivative obtained at DFT BP86 level of theory using TZVPP basis set.

In case of p-xylene derivative, tetrapyrazolyl ligands were obtained for both unsubstituted and 3,5-dimethylsubstituted pyrazole [13]. In contrast, o-xylene derivative reacted only with unsubstituted pyrazole, while in case of 3,5-dimethylpyrazole only starting materials were recovered, which is again due to steric reasons. Greater steric hindrance of compounds with o-phenylene spacer is seen from comparison of relative energies of compound **4** and its isomers and derivatives computed at DFT level (Table 1). As one can see from Table 1, energies of ortho-derivatives are higher than for the corresponding metaderivatives, and the energy difference is higher in case of dimethyl-substituted compounds. The greater steric hindrance of 3,5-dimethylsubstituted derivative of compound **4**, resulting from closely located methyl groups can be clearly seen from Figure 2, showing molecular models obtained and DFT BP86 level of theory.

The properties of pyrazole-containing ligands can be tuned by introducing electron-donating or withdrawing functional groups into the heterocyclic rings. One of these groups are iodine atoms, which are easily introduced into electron-reach pyrazole rings and can be exchanged to a variety of other groups by cross-coupling and substitution reactions. The oxidative system containing iodine and iodic acid in acetic and sulfuric acids used earlier by our group and others [14, 15] for pyrazole ring iodination proved unsuitable for compound **4** since it underwent acidic hydrolysis and only 4-iodopyrazole was isolated. However, using the modified procedure [16], involving dioxane water as solvent and excess of HIO_3_ instead of acetic and sulfuric acid allowed to obtain the tetraiodo derivative **5** in good yield.

## 4. Conclusion

In summary, we have prepared several new bitopic pyrazole-derived ligands that are interesting as building blocks for supramolecular chemistry and crystal engineering.

## Figures and Tables

**Table 1:** Relative energies of bis(bis(pyrazol-1yl)methyl)benzene derivatives.

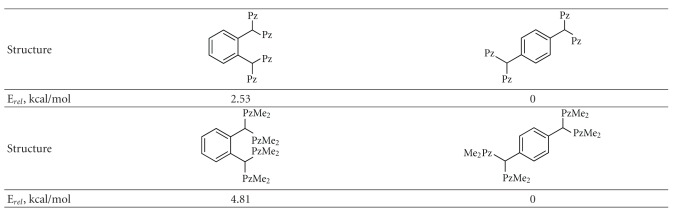

Notes: Pz = pyrazol-1-yl; PzMe_2_ = 3,5-dimethylpyrazol-1-yl; energies are computed at RI BP86 level of theory using TZVPP basis set.
